# To Screen or not to Screen: Low Dose Computed Tomography in Comparison to Chest Radiography or Usual Care in Reducing Morbidity and Mortality from Lung Cancer

**DOI:** 10.7759/cureus.589

**Published:** 2016-04-27

**Authors:** Joshua Dajac, Jay Kamdar, Austin Moats, Brenda Nguyen

**Affiliations:** 1 College of Medicine, University of Central Florida

**Keywords:** literature review, lung cancer, screening, low dose computed tomography

## Abstract

Lung cancer has the highest mortality rate of all cancers. This paper seeks to address the question: Can the mortality of lung cancer be decreased by screening with low-dose computerized tomography (LDCT) in higher risk patients compared to chest X-rays (CXR) or regular patient care? Currently, CXR screening is recommended for certain high-risk patients. Several recent trials have examined the effectiveness of LDCT versus chest radiography or usual care as a control. These trials include National Lung Screening Trial (NLST), Detection And screening of early lung cancer with Novel imaging TEchnology (DANTE), Lung Screening Study (LSS), Depiscan, Italian Lung (ITALUNG), and Dutch-Belgian Randomized Lung Cancer Screening Trial (Dutch acronym: NELSON study). NLST, the largest trial (n=53, 454), demonstrated a decrease in mortality from lung cancer in the LDCT group (RRR=20%, P=0.004). LSS demonstrated a greater sensitivity in detecting both early stage and any stage of lung cancer in comparison to traditional CXR. Although the DANTE trial yielded data consistent with findings in LSS, it also showed that via LDCT screening a greater proportion of patients were placed under unnecessary surgical procedures. The Depiscan trial yielded a high nodule detection rate at the cost of a high false-positive rate compared to CXR screening. The ITALUNG and NELSON trials demonstrated the early detection capabilities of LDCT for lung cancers compared to usual care without surveillance imaging. False-positive findings with unnecessary workup, intervention, and radiation exposure remain significant concerns for routine LDCT screening. However, current data suggests LDCT may provide a highly sensitive and specific means for detecting lung cancers and reducing mortality.

## Introduction and background

Currently, lung cancer has the highest mortality rates of all cancers in both men and women [1]. The estimated number of deaths from lung cancer in 2015 was 158,040 [1]. This may be attributed to the late stage of the disease at initial diagnosis. Discovering the lung cancer at an earlier stage that is more responsive to treatment could reduce the mortality; the 10-year survival rate for Stage I lung cancers has been estimated at 88% [2]. One example of the impact of effective screening tests can be seen with cervical cancer. Screening with Pap smears/HPV testing has remarkably reduced the incidence of and mortality from cervical cancer in the United States by more than 50% in the last 30 years [3]. A screening test for other deadly cancers have been long sought after, especially for the biggest killer in the US: lung cancer.

The USPSTF updated its recommendations on lung cancer screening in 2013 based on their systemic review on the effect of radiographic screening on lung cancer mortality. Adults age 55 to 80 with a 30 pack-year smoking history who either currently smoke or have quit within the past 15 years are candidates for annual LDCT screening for lung cancer. The recommendations exclude patients who have not smoked for 15 years or longer, or have a health problem that limits life expectancy or makes curative lung surgery impractical [4]. In the last few years, trials have studied the effectiveness of yearly LDCT scans in patients with more risks for lung cancer as a screening tool by comparing it to chest X-rays or regular patient care. This paper seeks to answer the following question with the evidence currently available: Can mortality from lung cancer be decreased by screening with LDCT scans in higher risk patients compared to chest X-rays or regular patient care?

Several trials have published their results thus far. The trials can be grouped based on their respective control groups: LDCT versus chest X-ray screening and LDCT versus no screening with regular patient care. The former consists of the following: National Lung Screening Trial (NLST), Detection And screening of early lung cancer with Novel imaging TEchnology (DANTE) trial, Lung Screening Study (LSS) trial, and the Depiscan trial. Two trials use regular patient care as the control group: the Italian Lung (ITALUNG) trial, and the Dutch-Belgian NELSON trial. This paper will look at the current results of each of these trials as well as a meta-analysis of current available research.

## Review

The NLST is by far the largest trial sporting an enrollment size of 53,454 [5]. 26,722 were randomly assigned to the group receiving three annual low-dose CT scans and 26,732 received annual posterioanterior chest X-rays. The participants had the following baseline prognostic characteristics: 55-74 years old at randomization (with 10 patients outside of this age range), 30 or more pack-years. The exclusion criteria were the following: quit smoking more than 15 years prior to enrollment, previous diagnosis of lung cancer, underwent chest CT within 18 months before enrollment, had hemoptysis, or unexplained weight loss of more than 6.8 kg in the last year. The patients were further stratified into five-year gaps, sex, race/ethnic group, and whether they are a current or former smoker. Each patient was followed and any findings on imaging, test/biopsy results, lung cancer diagnoses, and deaths were recorded. The patients who dropped out or did not receive at least one screening were not included in the analysis. The low-dose CT scan group had 97% of participants’ vital status known, and the control group had 96% [5].

This paper will focus on the results regarding lung cancer-specific mortality which are the following: 356 deaths from lung cancer in the low-dose CT scan group and 443 in the chest X-ray group. Thus the absolute risk reduction (ARR) = 0.3125% and the relative risk reduction (RRR) = 20% (95% CI, 6.8 to 26.7; P = 0.004), demonstrating a significant reduction of risk. The number needed to screen with low-dose CT scans to prevent one death from lung cancer = 320. Looking at the whole study demonstrates impressive numbers. The large power involved in the study (estimated 90% to detect a 21% decrease in mortality from lung cancer) combined with the RRR=20%, P=0.004, certainly lends itself to the potential utility of low-dose CT scans in high risk patients [5].

However, there are certainly adverse outcomes to screening and complications of diagnostic procedures to consider. The rate of at least one complication for a positive screening test was 1.4% for the low-dose CT scan group and 1.6% for the control. In the low-dose CT scan group, 16 patients (10 had lung cancer) died within 60 days of an invasive procedure and 10 (all had lung cancer) in the chest X-ray group. It cannot be determined with certainty that a complication of an interventional procedure was the cause of death in these cases. If that is the case, the low frequency shows that it rarely occurs and would be a significantly smaller number than the potential decrease in lung cancer-specific mortality discussed earlier. The NLST supports the use of annual low-dose CT scan to screen for lung cancer [5]. However, it is certainly worth looking at the other trials from Europe to come to a consensus with all of the results considered.

In the National Lung Screening Trial (NLST), LDCT was shown to reduce lung cancer mortality by 20% compared to traditional CXR using three rounds of CXR versus three annual rounds of LDCT. However, there is still uncertainty regarding efficacy and cost-effectiveness in a community setting. Additional trials such as the Prostate, Lung, Colorectal, and Ovarian cancer screening trial (PLCO) from 2011 reported no reduction of lung cancer mortality with CXR screening. The Early Lung Cancer Action Program (ELCAP) further supported the NLST conclusions via their own study, which established that LDCT has greater sensitivity than CXR for detecting small lung nodules. However, additional data from controlled trials were needed. The DANTE study was initiated in 2001 and completed in 2013 to address these concerns. This was the first and smallest of trials aimed at comparing lung cancer mortality with LDCT screening versus no screening [6].

The DANTE (Detection And screening of early lung cancer with Novel imaging TEchnology) trial compared the effect of LDCT screening on lung cancer mortality versus no screening. The primary endpoints were death from lung cancer and death from all other causes. The secondary endpoints were incidence, stage, and resectability rates of lung cancer. The subject of the study included only male smokers of 20+ pack years, age 60-74 years and who quit <10 years before trial. The subjects were recruited over a five-year time course and were divided among two arms: LDCT screening and control arm. A total of 1,264 people were enrolled in LDCT arm and 1,186 people were included in the control arm. In the screening arm, baseline CXR with sputum cytology and five screening rounds with LDCT were completed. In the control arm, yearly clinical review in randomized fashion was performed. The endpoints were obtained by obtaining death certificates and life status data for entire study population at the end of the study [6].

From this trial, the screening sensitivity for primary lung cancer detection using LDCT was 0.7952 and negative predictive value was 0.9813. About 37% in the screening arm had at least 1 abnormality by LDCT and 28% of the LDCT arm were further tested and received a total of 562 additional CT scans. A total of 104 subjects were diagnosed with lung cancer via screening (66 with CT, 1 by sputum cytology, 37 for other reasons) with 47 in Stage 1. Whereas, 72 people were diagnosed with lung cancer in the control group (ten with CXR and/or sputum at baseline, three at scheduled annual review, 59 due to symptoms) with 16 in Stage I. Both arms detected 50 cases of lung cancer at Stage II-IV. Overall, 32 more lung cancer patients were diagnosed in the screening arm in the face of unchanged mortality rate. In addition, significantly more patients with lung cancer were responsive to complete surgical resection in the LDCT group than control (7.12% vs 2.62%). Mortality was 543/100,000 person-years in LDCT versus 544/100,000 person-years in the control arm giving an ARR of 0.00001, RRR of 0.0018, and NNT of 544 [6]. 

Overall, DANTE had the highest proportion of subjects diagnosed with lung cancer among all trials and higher rate of surgical procedures. The DANTE trial detected Stage I lung cancer by CT in screened population more frequently than all other randomized trials. Significantly more invasive surgical procedures for potential cancer lesions were recorded in the LDCT arm with 18.9% undergoing surgery but did not reveal any cancer. This indicated that 1 out 5 patients, who were screened as a false positive, had undergone unnecessary surgery for a benign lesion [6].

The conclusions that were drawn from the DANTE trial were that DANTE cannot replicate results of the NLST trial due to multiple factors: small sample size, a randomized rather than observational trial, males-only study, control has baseline CXR + sputum exam + yearly clinical review (not reflecting community setting), and low sensitivity screening protocol. Lung cancer and all-cause mortality were similar in the screening and in the control arm. There was no definitive statement of efficacy of LDCT screening and the importance of obtaining additional data from randomized trials with intervention-free reference arms were needed before recommendations for population screening is possible [6].

LSS is a pilot study initiated in 2000 with the objective of assessing the feasibility of conducting a large scale randomized control trial (RCT) in comparing LDCT to traditional chest X-rays (CXR) for lung cancer screening. Chest X-rays were used as the control for this study because at the time the PLCO trial was still ongoing and their objective was to compare CXR to usual care. Therefore, by using CXR as the control, the results would be informative regardless of the outcome of PLCO trial. The initial LSS study created a baseline screening and the data was published, demonstrating that an RCT for lung cancer screening was plausible, which initiated the development of NLST. The scope of the initial LSS was further expanded to include a second screen one year following the initial baseline screening. The people recruited for the study had to meet the following requirements: 55-74 years old, 30 pack-year history of cigarette smoking, and current smoker or has quit ≤10 years ago. The subjects who were gathered at the end consisted of 59% male, 68% age 55-64 with 32% 65-74, 58% current smokers, and median of 54 pack years. Populations excluded from the study were patients who have received spiral CT of lungs/thorax in the previous 24 months, history of lung cancer, current treatment for any cancer other than non-melanoma skin cancer, total/partial pneumonectomy, and participation in another cancer screening trial/cancer primary prevention trial other than smoking cessation study. A total of 1660 subjects were randomized to the LDCT and 1658 into the CXR arm [7].

Compliance with screening declined in the LDCT arm from 96% to 86% at year one and CXR screening declined from 93% to 80%. The LDCT arm had lower screening compliance in subjects with positive baseline screens (77%) in comparison to subjects with negative baseline (91%). In this study, any non-calcified nodule >4 mm was a positive screen and considered suspicious for lung cancer. All subjects with positive screen were referred to personal health care providers for diagnostic follow-up. LSS did not specify the diagnostic algorithm and lung cancer was diagnosed via tracking medical records. Moreover, patients who were tested positive at baseline and were later diagnosed with lung cancer were not included for the year one screen [7].

LSS demonstrated positivity rates 25.8% for LDCT and 8.7% for CXR in LSS trial; positivity rates in similar studies varied possibly due to differences in definition of a positive screen. In the study, each subject was monitored individually for the presence and size of nodules at baseline versus year one LDCT screen. Cancer detection yield was less at year one for LDCT at 0.57% versus baseline of 1.9% whereas CXR increased from 0.45% at baseline to 0.68% at year one. Twice as many lung cancer diagnoses were made in the LDCT arm compared to CXR arm. In both arms, subjects with positive baseline screens were much more likely to have positive screens at year one than subjects with negative baseline screens. Based on the data from the study, 42% of LDCT and 30% of CXR arm that were positive at year one had a positive baseline. Follow-up rates with personal healthcare with positive LDCT at year one (40%) was lower than with positive baseline LDCT screen (73%). Overall, 40 lung cancers were diagnosed in LDCT arm and 20 detected in CXR arm. Of the lung cancers diagnosed, LDCT detected 48% of Stage 1 cancers and CXR arm detected 40%. Stage III-IV cancers, on the other hand, were detected in 40% of LDCT arm versus 45% in CXR arm. Absolute number of Stage III-IV cases were greater in LDCT than CXR. From the successful completion of LSS, it established the feasibility of an RCT comparing annual spiral CT to CXR for lung cancer screening and implicated what should be expected regarding screening compliance, screen positivity, diagnostic follow-up, and lung cancer yield for NLST [7].

The Depiscan trial was designed with the intervention of LDCT and the control arm was chest X-ray. Imaging was obtained at baseline and annually for two years. The inclusion criteria included age 50-75 years, asymptomatic current or former smokers (less than 15 years from enrollment), cigarette consumption greater than or equal to 15 cigarettes per day for at least 20 years. The exclusion criteria included previous history of malignancy, having contraindication for imaging or previous history of lung or heart disease [8].

Seven-hundred sixty-five participants were randomized with 385 participants in the intervention arm of LDCT and 380 participants in the control arm of chest X-ray. However, due to refusal of LDCT or chest X-ray, 336 were in the LDCT group and 285 were in the chest X-ray group. The trial was 72% male with a median age of 56 (47-76). 64% were current smokers and had one median pack of cigarettes per day and a 32-year smoking history. 36% were former smokers and had one median pack of cigarettes per day and a 30-year smoking history [8].

LDCT was able to detect more nodules than the chest X-ray. One-hundred fifty two nodules were found in the LDCT group while only 21 were found in the chest X-ray group. LDCT was statistically significant compared to chest X-ray for detecting nodules, 10.42 odds ratio (CI 95%, 6.36 – 17.07). For every category of nodule size, less than 5 mm, 5 to 10 mm, greater than 10 mm, LDCT was able to detect a higher percentage than chest X-ray. For nodules less than 5 mm, LDCT detected 71 while chest X-ray detected six. For nodules 5 to 10 mm, LDCT detected 53 while chest X-ray detected eight. For nodules greater than 10 mm, LDCT detected 28 while chest X-ray detected seven. LDCT detected eight nodules that turned out to be lung cancer while chest X-ray could only detect one [8].

LDCT was able to detect almost 10 (6.36-17.07) times the amount of non-calcified nodules than a chest X-ray. Even though almost 152 nodules were found using LDCT, only eight of them were lung cancer. This would lead to a high false positive rate. Three unnecessary thoracotomies were performed because of false positives. Multiple LDCTs leads to increased radiation. The effect of radiation from multiple LDCTs cannot be ignored and must be balanced with the benefit of early detection of lung cancer. The balance between the benefits and risks of LDCTs is still being studied and a decision at this time cannot be made. The 10-year survival rate for LDCT detected lung cancer is 80% while the Stage I lung cancer rate is 88%. By detecting more cancers when they are Stage I, the patient survival rate will increase leading to better patient outcomes [8].

The Italian Lung (ITALUNG) study was a small scale RCT component of the EU-US Collaborative Spiral CT working group aimed to determine differences in lung cancer mortality between high-risk patients receiving annual LDCT screening and those undergoing usual care. Specifically, 3206 subjects from Tuscany, Italy were assigned randomly to the active arm (1613) and control arm (1593). Patients were between 55 and 69 years old who smoked at least 20 pack-years within the last 10 years. Exclusion criteria included a history of previous cancer except for non-melanoma skin cancer as well as general conditions requiring thoracic surgery. The active arm (1406 after 207 dropout prior to baseline CT) underwent LDCT screening annually for four years; the control arm received usual care without any radiological screening [9].

At the conclusion of the trial, only 15.7% of patients in the active arm had a positive screening LDCT. Thirty-eight patients were diagnosed with lung cancers with 20 at initial baseline screening and 18 during annual screening. The study had a lung cancer prevalence at baseline screen of 1.5% and a mean incident cancer detection rate of 0.5%, values comparable to other RCTs and observational studies. Despite the relatively low detection rate, the study protocol for follow-up LDCT with PET scans, fiber optic bronchoscopy, and fine needle aspiration biopsy for positive screens yielded a 100% sensitivity and only a 10% surgery rate for benign lesions. Perhaps most importantly, the majority of patients with non-small cell lung cancers (NSCLC) detected by LDCT (23 out of 35 patients) were identified at Stage I [10].

The study was not without its limitations. As a small scale study within a small region of Italy results are not necessarily comparable to the general population. Furthermore, the method of recruitment by direct mail out had a 12.8% dropout rate, much higher than the NLST (2.6%) and Danish RCT (0.2%). The study also had a high recall rate; 52.7% of patients in the active arm were recalled at least once for follow-up despite only 2.7% of patients in the active arm having a final cancer diagnosis [10]. Nonetheless, preliminary results bode well for future survival analysis and the combined results by the EU-US Collaborative Spiral CT Working Group in support of LDCT screening.

The Dutch-Belgian lung cancer screening trial NELSON is currently the largest European trial evaluating the potential for LDCT as a screen for lung cancer. The study assessed whether increasing intervals between LDCT screens could reduce mortality from lung cancer when compared to usual care without screening. A total of 15,822 patients from the Netherlands and Belgium were randomized to the LDCT screening group (7915) or the control group (7907). The patients were between age 50-75 years old with smoking histories of either A) 15 or more cigarettes per day for more than 25 years or B) 10 or more cigarettes per day for more than 30 years. In addition, patients were current smokers or had smoked within the last 10 years. Exclusion criteria included self-reported moderate to bad health; the inability to climb two flights of stairs, body weight of 140 kg or greater; history of renal cancer, melanoma, or breast cancer; a lung cancer diagnosis made within the last five years; and a chest CT conducted within the last year. The screening group underwent baseline LDCT with three subsequent screens made at 1, 3, and 5.5 years after baseline screen, representing 1, 2, and 2.5 year intervals, respectively [11].

A total of 463 patients had a final positive screening LDCT. Of these patients, 187 were diagnosed with 196 lung cancers. The overall positive predictive value for screening was 40.4% (95% CI, 35.9-44.7), significantly greater than that of the NLST at 3.8% (95% CI, 3.4-4.3) and other European studies. Sensitivity for round one of LDCT one year after baseline screen was 92.5% (95% CI, 85.5-98.4), comparable to 93.8% (95% CI, 90.6-96.3) for the NLST. There was a slight drop off in sensitivity with the two-yearly screening to 73.6% (95% CI, 62.5-83.6); specificity remained high at 99.0% (95% CI, 98.8-99.2). Sensitivity was comparable to the Italian Multicentric Italian Lung Detection (MILD) study assessing two-yearly interval LDCT screening (80.0%). Similar to the ITALUNG and NLST studies, NELSON detected a majority of lung cancers at early stages; 62% of cancers from positive screens were Stage I compared to only 18% detected at stage IIIB or IV [11].

Unfortunately, the analysis reviewed here excluded the Belgian population as the researchers did not have access to the Belgian cancer registry data. The number of participants and therefore power of the study are also much smaller than that of the NLST. However, the comparable sensitivity, specificity, positive predictive value, and early detection for LDCT in the NELSON study to larger scale RCTs provides significant data for a future standardized lung cancer screening protocol. In addition, initial data shows that biannual screening may be as effective as annual screening, reducing costs and patient radiation exposure [11]. Further study will be conducted using mortality analysis 10 years after initial randomization.

In order to better quantify the benefit and risks of low dose CT versus a control (chest X-ray or usual care), a systematic review was performed by Dr. Gopal and Dr. Abdullah. This review included randomized controlled trials published between 1966 and February 2010. These were acquired using the search concepts “lung neoplasm,” “mass screening,” “CT,” and “X-ray. The participants in the studies were all high risk for lung cancer, which includes being older (average age was 50-60), and having a longer pack year history of smoking, average pack year history was 20-30 pack years. The intervention for all the studies was low dose CT while the control was either no screening other than usual care in three studies and chest X-ray in the three other studies. The outcomes that were measured were Stage I non-small cell lung cancer, any kind of stage non-small cell lung cancer, any kind of lung cancer, false-positive nodules (benign non-calcified nodules that were greater than 4 mm), and rate of thoracotomy for benign lesions. No studies were excluded because of quality issues [12].

There were a total of 14,055 participants combined for all six studies and 7078 of those participants were in the LDCT arm while 6977 were in the control arm. For the six studies, baseline characteristics were collected including age, gender, smoking history, length of the screen after the intervention or control, and type of intervention. The age range of the participants for all six studies were from 49-80 while the narrowest range was 55-69 in the ITALUNG study and the widest range was 50-80 in the Garg/Colorado University study. The gender percentage ranged from 100% male in the DANTE trial to 55.2% male in the DANISH trial. For all six studies, the participants’ smoking history was greater than 15 years and all but in the DEPISCAN trial it was greater than 20 years smoking history. Ex-smokers all quit at least 10 years before the study began. Length of screening were at least one year for all studies and most of them were at least two years. As stated before, all six study interventions were LDCT but three of the studies had a control of usual care while three others had a chest X-ray [12].

Even though all six studies were similar in their participants and intervention, there were a few differences. The workup of the nodules detected on LDCT varied slightly, however, the diagnoses were the same such as benign nodules, Stage I NSCLC, or lung tumor. The follow-up was also slightly different with some of the trials having different frequencies in the screening regiment. Three studies used standard of care practices as a control while three others used chest X-ray. In order to compare both type of studies, meta-analysis was performed and it showed no statistically significant differences [12].

In order to compare the endpoints between the six different trials, odds ratios were used. Three main endpoints were compared between trials, number of Stage I NSCLC, total number of NSCLC, and false-positives. For all three endpoints, the positive results from LDCT compared to the control arm was statistically significant. For the number of Stage I NSCLC, the odds ratio is 3.905 (95% CI, 2.052–7.430). The Garg and DEPISCAN trials were the only ones that were not statistically significant with an odds ratio of 3.230 (95% CI, 0.130–80.283) and 6.963 (95% CI, 0.358–135.267) respectively. The other four trials had an odds ratio of 2.680–20.869 with the lower limit of the 95% CI greater than 1.110 in all trials and the upper limit of the 95% CI greater than 6.865 [12].

For the number of total NSCLC, the odds ratio is 5.507 (95% CI, 3.127–9.698). The Garg trials was the only one that was not statistically significant with an odds ratio of 3.230 (95% CI, 0.130–80.283). The other five trials had an odds ratio of 4.194–36.954 with the lower limit of the 95% CI greater than 1.001 in all trials and the upper limit of the 95% CI greater than 9.600 [12].

For the number of false-positives nodules, the odds ratio of 3.122 (95% CI, 2.621–3.720). All six trials were statistically significant and an odds ratio of 2.255–1980.070 with the lower limit of the 95% CI greater than 1.825 in all trials and the upper limit of the 95% CI greater than 2.787. The ITALUNG trial skewed the results with an odds ratio of 1980.070 (95% CI, 123.573–31727.701) [12].

Compared to participants who were in the control arm, participants who were in the LDCT arm were more likely to undergo a thoracotomy for a benign lesion. All six studies were statistically significant and most of them were significant at an alpha level of 0.01 instead of the usual 0.05. The event rate for thoracotomy was 0.371 for all six studies combined [12].

Early detection of lung cancer leads to a better prognosis, however, for many patients, lung cancer is asymptomatic during the early phase of disease. Several screening tools including chest X-ray and sputum cytology have shown no benefit and actually leads to a higher false-positive rate. The advances in CT technology including low dose CT led people to believe that LDCT can be used as a screening tool for high risk patients. Previous studies have shown that CT can pick up eight times more cancers than chest X-rays. There are nine randomized clinical trials worldwide that test the effectiveness of screening in early detection for lung cancers. The six used in this meta-analysis include Garg/Colorado University, ITALUNG, NLS/LSS, DEPISCAN, DANTE and Danish [12].

Being able to detect lung cancers when they are Stage I leads to better prognosis and a higher five-year survival rate. Only 16% of lung cancers during routine care are Stage I but using LDCT, the rate is 70%, more than 3.9 times as evident in the six studies. However, screening with LDCT also has its risks. Overdiagnosis, detection of false-positive lesions and need for further work-up must be balanced with the increased detection of stage I NSCLC. Individuals are more likely, 3.1 times, to have false positive nodule with LDCT screening than the control arm [12].

The only limitations with this meta-analysis were the inherent problems with meta-analysis and heterogeneity of the studies. The NELSON randomized controlled trial was not available at the time of publication. In addition, each study had a slightly different protocol for the work-up of the nodules and the follow-up CT scans [12].

## Conclusions

This review discussed the results NLST, DANTE, LSS, Depiscan, ITALUNG, and NELSON trials in evaluating low dose CT as a plausible screening tool for lung cancer. Though the percentage of positive LDCT screening ranged between 5% and 35% in the studies, most studies demonstrated that LDCT had strong sensitivity and specificity in detecting lung cancer [5-12]. When compared to CXR surveillance, LDCT had a greater rate of lung cancer detection in the NLST, LSS, and Depiscan studies [5,7-8]. The number needed to screen with low-dose CT to prevent one death from lung cancer as estimated by the NLST was 320 [5], comparable or better than estimated values for currently employed screenings such as fecal occult blood screens for colon cancer and mammography for breast cancer [13]. The currently available mortality data from the studies shows mixed results. The NLST clearly supports the use of LDCT, estimating an up-to-21% reduction in lung cancer mortality compared to annual CXR screening [5]. However, the DANTE trial did not show a survival benefit for patients undergoing LDCT compared to CXR screening [6].

The studies reported relatively low positive predictive values for detecting lung cancer with none being greater than 50% [5-11]. The rate of false-positives and ensuing invasive workup and interventions remain a concern should LDCT be used as a screening tool. The DANTE trial brought up the most questions regarding complications from false-positives; 18.9% of patients who underwent surgery after positive screening were found to have benign lesions [6]. According to the meta-analysis by Gopal et al., for every 1000 individuals, nine Stage I NSCLC will be detected while 235 false-positive nodules will also be detected [12]. Four thoracotomies will be performed for benign lesions [12]. In addition to unnecessary interventions, LDCT exposes patients to greater radiation than traditional chest radiography. Due to the limited survival data available, it is difficult at this point to definitively evaluate whether the risks from excess radiation exposure and false-positive lesion workup are worth the modest cancer detection rates and predicted reduction in mortality.

Despite the issues with false positives, early lesion detection by LDCT may play a significant role in mortality reduction and prognosis. The ITALUNG and NELSON trials most notably showed high rates of detection of early stage lesions [9-11], which may correlate with mortality reduction in these patient pools. Though the results of LDCT screening are promising, researchers must optimize the frequency and length of screening to reduce overdiagnosis and other risks of LDCT.
